# Inhibition of cyclophilin A suppresses H_2_O_2_‐enhanced replication of HCMV through the p38 MAPK signaling pathway

**DOI:** 10.1002/2211-5463.12105

**Published:** 2016-08-15

**Authors:** Jun Xiao, Xin Song, Jiang Deng, Liping Lv, Ping Ma, Bo Gao, Xipeng Zhou, Yanyu Zhang, Jinbo Xu

**Affiliations:** ^1^ Beijing Institute of Transfusion MedicineChina; ^2^ Beijing Key Laboratory of Blood Safety and Supply TechnologiesChina

**Keywords:** cyclophilin A, cytomegalovirus, H_2_O_2_, p38 MAPK, viral replication

## Abstract

Human cytomegalovirus (HCMV) infection can be accelerated by intracellular and extracellular hydrogen peroxide (H_2_O_2_) stimulation, mediated by the activation of the p38 mitogen‐activated protein kinase (MAPK) pathway. However, it remains unknown whether host gene expression is involved in H_2_O_2_‐upregulated HCMV replication. Here, we show that the expression of the host gene, cyclophilin A (CyPA), could be facilitated by treatment with H_2_O_2_ in a dose‐dependent manner. Experiments with CyPA‐specific siRNA, or with cyclosporine A, an inhibitor of CyPA, confirmed that H_2_O_2_‐mediated upregulation of HCMV replication is specifically mediated by upregulation of CyPA expression. Furthermore, depletion or inhibition of CyPA reduced H_2_O_2_‐induced p38 activation, consistent with that of H_2_O_2_‐upregulated HCMV lytic replication. These results show that H_2_O_2_ is capable of activating ROS‐CyPA–p38 MAPK interactions to enhance HCMV replication.

**Abbreviations**
CsA: cyclosporine A
CyPA: cyclophilin A
H_2_DCFDA: 2′,7′‐dichlorodihydrofluorescein diacetate
HCMV: human cytomegalovirus
HDACs: histone deacetylases
HFF: human foreskin fibroblast
IE1: immediate early
MAPK: mitogen‐activated protein kinase
MEF: mouse embryonic fibroblast
MOI: multiplicity of infection
NAC: *N*‐acetylcysteine
PPIase: peptidylprolyl‐*cis–trans*‐isomerase
ROS: reactive oxygen species
TCID_50_: 50% tissue culture infective dose
VSMC: vascular smooth muscle cells

Human cytomegalovirus (HCMV) is a widespread pathogen that establishes a lifelong persistent infection and causes life‐threatening symptoms in immunocompromised or immunosuppressed hosts 1. There are three drugs currently approved for HCMV treatment: ganciclovir and its prodrug valganciclovir, foscarnet, and cidofovir 2. However, the utility of each has been limited by significant toxicity. Since combinatorial treatments are not currently being considered due to their cytotoxicity, therapies with diverse mechanisms of action are highly desirable. Earlier work from our laboratory 3 and others 4, 5 has demonstrated that oxidative stress could upregulate HCMV replication, and identify antioxidants as a possible target for treatment of HCMV infection. However, the underlying mechanism linking oxidative stress and HCMV replication remains poorly characterized.

Cyclophilin A (CyPA) was originally discovered as a cellular factor with high affinity for the immunosuppressant cyclosporine A (CsA) 6. Previous studies have demonstrated that ischemia/reperfusion (I/R) and hypoxia can induce expression of CyPA 7, 8, 9. CyPA can be also secreted from monocytes/macrophages 10, endothelial cells 11, and vascular smooth muscle cells 12 in response to ROS. Elevated CyPA have been reported in association with various viral infections. Studies have shown that HIV‐1 replication was reduced in human CD4+ T cells when CyPA was knocked out 13. Thus, these studies suggest CyPA plays a significant role in promoting HIV infection. CyPA was reported to bind HIV‐1 Gag polyprotein in a Cs‐sensitive complex 14, which is essential for HIV replication 15. Recent studies have reported that CypA induces a repressive effect on the replication of some viruses, including influenza A virus 16, rotavirus 17, and HEV 18.

CyPA has also now been understood to represent a key factor in the regulation of cytomegaloviruses, modulating replication of murine cytomegalovirus (MCMV) and HCMV 19, 20. During MCMV infection in NSPC (Neural stem/progenitor cells), CyPA has been suggested to play an important role in regulation of major immediately early promoter (MIEP) chromatin modification by interacting with histone deacetylases (HDACs). Despite the enormous potential for CyPA in modulating viral response, the role of CyPA during ROS‐upregulated HCMV replication remains largely uncharacterized. In this study, we examined whether CyPA participates in hydrogen peroxide (H_2_O_2_)‐mediated cytomegalovirus (CMV) replication and its mode of action.

## Materials and methods

### Cell culture, chemical reagents and antibodies

Human foreskin fibroblast (HFF) cells, mouse embryonic fibroblast (MEF) cells of no more than 15 passages, and HEK 293 cells were cultured in Dulbecco's modified Eagle's Medium (DMEM) supplemented with 10% FBS at 37 °C under a 5% CO_2_ atmosphere 3.

H_2_O_2_ solution, *N*‐acetylcysteine (NAC), CsA, 2′,7′‐dichlorodihydrofluorescein diacetate (H_2_DCF‐DA), *N*‐succinyl‐Ala‐Ala‐Pro‐*p*‐nitroanilide, α‐Chymotrypsin, and the p38 inhibitor SB203580 were purchased from Sigma Life Science (St. Louis, MO, USA).

Rabbit polyclonal or monoclonal antibodies used in this study included phospho‐p38 (T180/Y182), p38, CyPA, and β‐actin were provided by ABclonal technology (Cambridge, MA, USA) and the mouse monoclonal antibodies to HCMV pp72 and pp65 were purchased from Santa Cruz Biotechnology (Santa Cruz, CA, USA) 3.

### Viral preparation and titration

Human cytomegalovirus (AD169strain) and MCMV (Smith strain) stocks were prepared in HFF cells and MEF cells, and aliquots were stored at −80 °C. HCMV was used to infect cells at a multiplicity of infection (MOI) of 0.5 for all of the current experiments. Viral titers were detected using the 50% tissue culture infective dose (TCID_50_) assay, as previously described 3, 21. All experiments were examined at least three times using Reed and Muench's method.

### Generating stable CyPA knockdown cell lines

Lentiviral vectors, targeting cyclophilin A (siCypA) or a random sequence (siCTR), were produced by Hanbio Co. Ltd (Shanghai, China). The siRNA sequences were: CyPA, 5′‐GATCCGTGGTGACTTCACACGCCATAATTCAAGAGATTATGGCGTGTGAAGTCACCATTTTTTC‐3′. HFF and HEK293 cells were infected at an MOI of 100 or 10, respectively. The siRNA recombinant lentivirus was incubated with 8 μg·mL^−1^ polybrene to enhance the lentivirus infection. For the stable knockdown cell lines, the HFF or HEK293 cells were incubated in a selection medium containing 2 μg·mL^−1^ puromycin (Invitrogen, Carlsbad, CA, USA) beginning 48 h after transduction.

### Dichlorofluorescein staining

Dichlorofluorescein staining was operated as previously described with slight modification 3. Cells were seeded on 24‐well culture plate, stimulated with H_2_O_2_ (200 μm) for 24 h or infected with MCMV for 72 h and then incubated with H_2_DCF‐DA (10 μm) in serum‐free DMEM for 0.5 h at 37 °C (dark conditions). Cells were then washed with PBS three times and images were taken by Leica microscope (Wetzlar, Germany).

### Luciferase assays

In 24‐well plates, 10^5^ cells per well were cultured to confluence and were transiently transfected with the MIEP‐pGL3 reporter plasmid and the pRL‐TK vector. Twelve hours following transfection, the cells were treated with the CsA (1 μm) for 1 h and then stimulated with H_2_O_2_ (200 μm) for 12 h. Luciferase activity was measured as previously described 3, 22 using a Dual‐Luciferase^®^ Reporter Assay System (Promega, Madison, WI, USA).

### Western blot analysis

Western blot analysis was carried out as previously described 23. Proteins in cell lysates were heated for 5 min at 95 °C, and loaded onto a 12% SDS/PAGE gel. Proteins were separated by electrophoresis and transferred onto polyvinylidene difluoride (PVDF) membranes (Millipore, Billerica, MA, USA), and incubated with indicated antibodies. Proteins bands were visualized using western blotting luminol reagent according to the manufacturer's protocol (Santa Cruz Biotechnology). The membranes were incubated with western blot stripping buffer (CWBio, Beijing, China) to reprobe for other proteins on the same membrane.

### CMV genome quantification

Total DNA was isolated as previously described 3. Viral DNA was quantified using quantitative PCR (qPCR) on a CFX‐96 thermocycler (Bio‐Rad, Hercules, CA, USA), using CMV specific primers. Primers used are 5′‐ATGTACGGGGGCATCTCTCT‐3′ (forward) and 5′‐GGCTTGGTTATCAGAGGCCG‐3′ (reverse) for HCMV genome or the MCMV genomic primer, 5′‐GTGGGCATGAAGTGTGGGTA‐3′ (forward) and 5′‐CGCATCGAAAGACAACGCAA‐3′ (reverse).

### Real‐time quantitative PCR

The real‐time quantitative PCR (RT‐qPCR) method was described previously 3. Briefly, total RNA was extracted using TRIzol reagent (Invitrogen) 24 h following HCMV infection (MOI = 0.5). Approximately 500 ng of RNA was transcribed into cDNA using ReverTra Ace^®^ qRCR RT Master Mix with gDNA Remover (TOYOBO, Osaka, Japan). Each sample was measured in triplicate. The HCMV IE1 expression level (forward primer, 5′‐GTTGGCCGAAGAATCCCTCA‐3′ and reverse primer, 5′‐CACCATGTCCACTCGAACCT‐3′) and human CyPA gene transcript (forward primer, 5′‐GCTGGACCCAACACAAATGG‐3′ and reverse primer, 5′‐GCTCCATGGCCTCCACAATA‐3′) were normalized to GAPDH mRNA (forward primer, 5′‐CATGAGAAGTATGACAACAGCCT‐3′ and reverse primer, 5′‐AGTCCTTCCACGATACCAAAGT‐3′). The expression of mouse CyPA gene transcript (forward primer, 5′‐AAAGCATACAGGTCCTGGCATC‐3′ and reverse primer, 5′‐CATGCTTGCCATCCAGCCAT‐3′) was normalized to mouse GAPDH mRNA (forward primer, 5′‐CCGTCGTGGATCTGACGTG‐3′ and reverse primer, 5′‐GGTCCTCAGTGTAGCCCAAG‐3′). Compared to the untreated cells or uninfected mice, the relative expression levels in treated cells and infected mice were calculated as fold changes.

### Animal studies

BALB/c mice (male, 3–4 weeks old, and 15–20 g body weight) were purchased from Vital River (Beijing, China). The protocols used in this study were approved by the Ethics Committee at the Beijing Institute of Transfusion Medicine and were performed in accordance with Institutional Animal Care and Use Committee (IACUC) guidelines.

Mice were treated with vehicle (olive oil) or 10 mg·kg^−1^·day^−1^ CsA by gavage, from 3 days before intraperitoneal inoculation with MCMV (Smith strain, 5 × 10^3^ p.f.u). At day 7, 14, 21 and 28 post infection, total DNA was extracted from 100 μL whole blood and used to detect the viral DNA genome. To measure infectious virions in mice organs, the salivary glands (50 mg) and the lung (50 mg) were collected on day 14 and 28 post infection and homogenized, then viral titer was calculated with TCID_50_ assays in MEF monolayers.

### CyPA activity assay

The CyPA activity was detected as previously described 24. The *cis*–*trans* isomerization of Ala‐Pro peptide bond in the test peptide *N*‐succinyl‐Ala‐Ala‐Pro‐*p*‐nitroanilide (100 μm) was measured in an assay with α‐Chymotrypsin (10 μm). Briefly, reactions were at 15 °C and contained 30 μL of test peptide, 0.1 m Tris‐HCl (pH 7.8) and 100 μg test sample. After incubation for 1 min, 30 μL of 2 mg·mL^−1^ α‐Chymotrypsin in 0.1 m Tris‐HCl was added. After mixing, the absorbance was detected at 390 nm.

### Statistical analyses

Statistical analyses were carried out using previously described methods 3. All values are expressed as the means ± standard deviations. Statistical analyses were performed using spss statistical software V.17 (SPSS Inc., Chicago, IL, USA). Significant differences were evaluated by two‐tailed Student's *t*‐test when two groups were compared, one‐way analysis of variance (ANOVA) followed by the Dunnett's test when multiple groups were tested against a control group and the Bonferroni *post hoc* test when performing multiple comparisons between groups. A *P*‐value less than 0.05 was considered as a statistically significant difference.

## Results

### H_2_O_2_ induces upregulation of cyclophilin A

**Figure 1:**
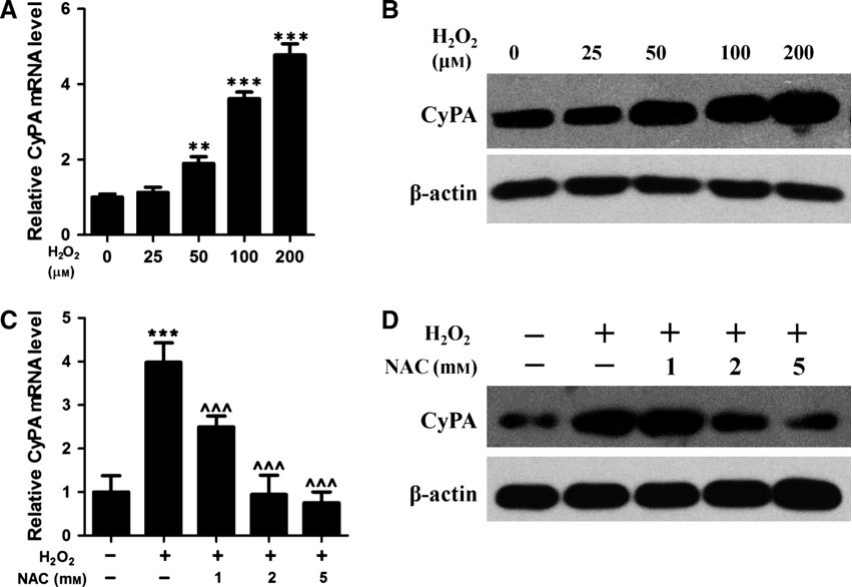
Cyclophilin A (CyPA) was induced in human fibroblast cells treated with H_2_O_2_. After supplementation with 0, 25, 50, 100, 200 μm H_2_O_2_ for 24 h, increasing CyPA levels in human foreskin fibroblast (HFF) cells were observed by RT‐qPCR (A) or by western blotting (B). Treatment of HFF cells with 0, 1, 2, 5 mm H_2_O_2_ scavenger *N*‐acetylcysteine (NAC) prior to H_2_O_2_ (200 μm) inhibited the H_2_O_2_‐induced CyPA expression (C) and protein changes (D). The data are expressed as the means ± SD. **P < 0.01 or ***P < 0.001 compared with control HFF cells and ^^^*P* < 0.001 versus H_2_O_2_‐treated cells.

To investigate whether CyPA can be enhanced in HFF cells by H_2_O_2_ stimulation, RT‐qPCR analysis was performed using mRNA extracted from HFF cells exposed to H_2_O_2_. Firstly, the effect of H_2_O_2_ on cell death was detected by MTT (3‐[4, 5‐dimethylthiazol‐2‐yl]‐2, 5‐diphenyl tetrazolium bromide) assay (Fig. S1). Then, an increase in CyPA transcripts levels were observed at 50 μm H_2_O_2_ and continued to increase up to a concentration of 200 μm (Fig. 1A). These findings were also recapitulated at the protein level (Fig. 1B). To confirm these results, we repeated the analysis using an antioxidant, *N*‐acetylcysteine (NAC), to counteract the effects of H_2_O_2_. Although increasing CyPA mRNA and protein levels were observed following exposure to 200 μm H_2_O_2_ (Fig. 1C,D), a significant decrease in CyPA expression was detected in a dose‐dependent manner following treatment with NAC (Fig. 1C,D). These results indicated that H_2_O_2_ could enhance the expression of CyPA in a dose‐dependent manner in HFF cells.

### Silencing CyPA in HFF cells prevents H_2_O_2_‐upregulated HCMV replication

**Figure 2:**
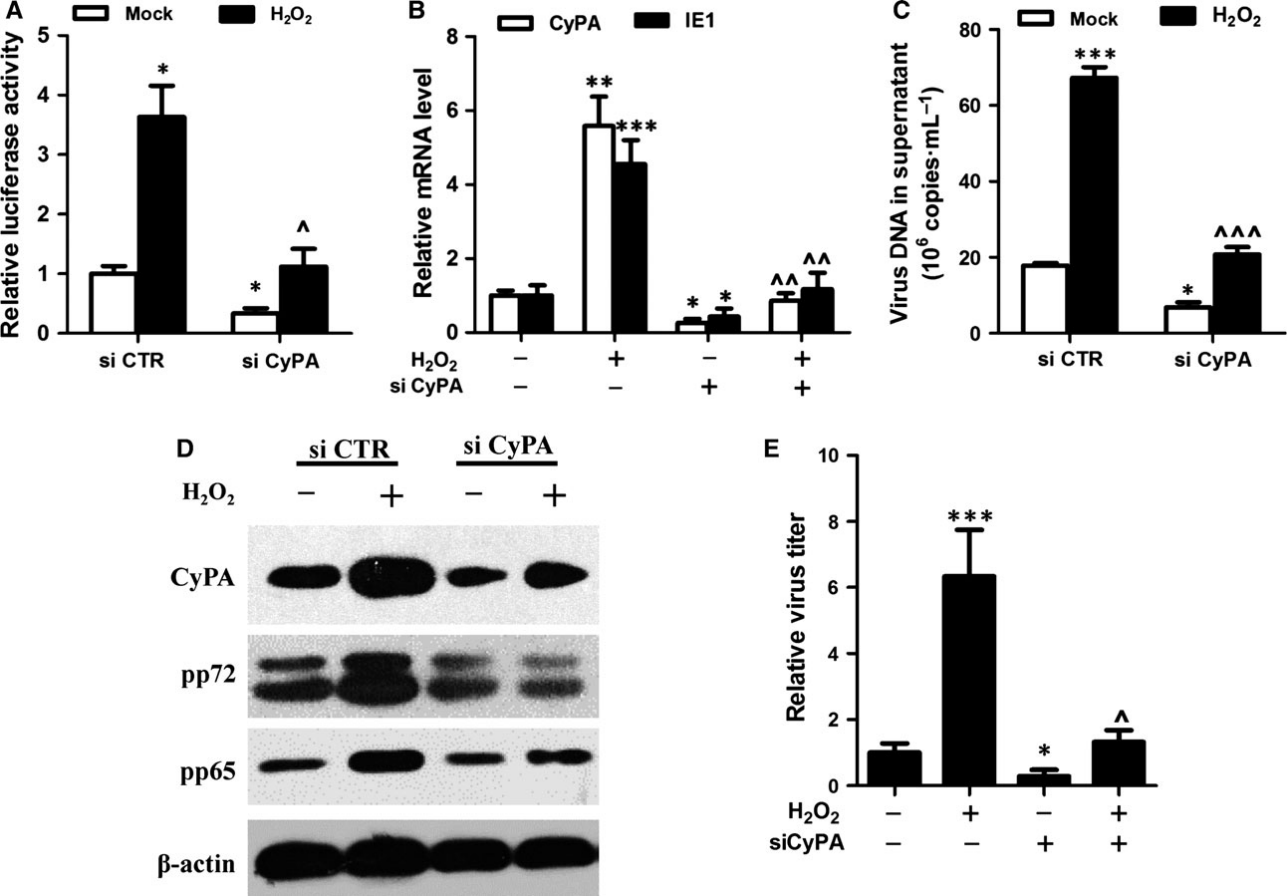
Depletion of CyPA protein delayed H_2_O_2_‐upregulated viral replication. Luciferase activities were measured in siRNA‐293 cells, stably expressing siCTR or siCyPA. These cells were transfected with MIEP‐pGL3 plasmid for 24 h and treated with 200 μm H_2_O_2_ for 12 h (A). Human foreskin fibroblast (HFF) siCTR and siCyPA cells were infected at a Multiplicity of Infection (MOI) of 0.5 for 24 or 72 h. Treated with 200 μm H_2_O_2_ for 24 h upregulated CyPA and human cytomegalovirus (HCMV) IE1 transcription in HFF siCTR cells, but the effect was attenuated in HFF siCyPA cells (B). The virions in the cultural supernatant and pp72 and pp65 viral proteins in HCMV infected (MOI = 0.5) HFF siCTR and siCyPA cells treated with H_2_O_2_ (200 μm) for 72 h were determined by qPCR and western blotting (C, D). HFF siCTR and siCyPA cells were infected with HCMV (MOI = 0.5) in the presence of H_2_O_2_ (200 μm) for 5 days, and monolayers were analyzed by viral titration (E). *P < 0.05; **P < 0.01 or ***P < 0.001 for treated versus mock treated cells or HFF siCTR cells. ^*P* < 0.05; ^^*P* < 0.01 or ^^^*P* < 0.001 for HFF siCyPA versus HFF siCTR under the treatment with H_2_O_2_.

CyPA has been known to play an important role in viral replication 25. To assess the effect of the CyPA on H_2_O_2_‐enhanced HCMV replication, we performed a luciferase assay to investigate the activation of HCMV MIE promoter after cells were stably transfected with siCTR or siCyPA and exposed to H_2_O_2_ for up for 12 h. Before H_2_O_2_ treatment, the baseline expression levels of CyPA were evaluated in transfected cells by western blotting. Compared with wild‐type cells, the expression of CyPA was reduced by approximately 90% following siCyPA interference (Fig. S2). CyPA knockdown resulted in a reduction of luciferase activity as compared to control cells in the context of H_2_O_2_, suggesting that CyPA is involved in H_2_O_2_‐upregulated MIEP activity (Fig. 2A). Next, we tested whether HCMV gene transcripts and protein expression were affected by CyPA interference in HFF. Consistent with the luciferase assay results, silencing of CyPA resulted in downregulation of H_2_O_2_‐enhanced CyPA and HCMV IE1 expression (Fig. 2B) and HCMV replication (Fig. 2C,D). Furthermore, the production of infectious virions was elevated with H_2_O_2_ in siCTR HFF cells while depletion of CyPA reversed this effect (Fig. 2E). These results indicate that CyPA plays an important in H_2_O_2_‐upregulated HCMV replication.

### The activity of CyPA is required for H_2_O_2_‐upregulated HCMV replication

**Figure 3:**
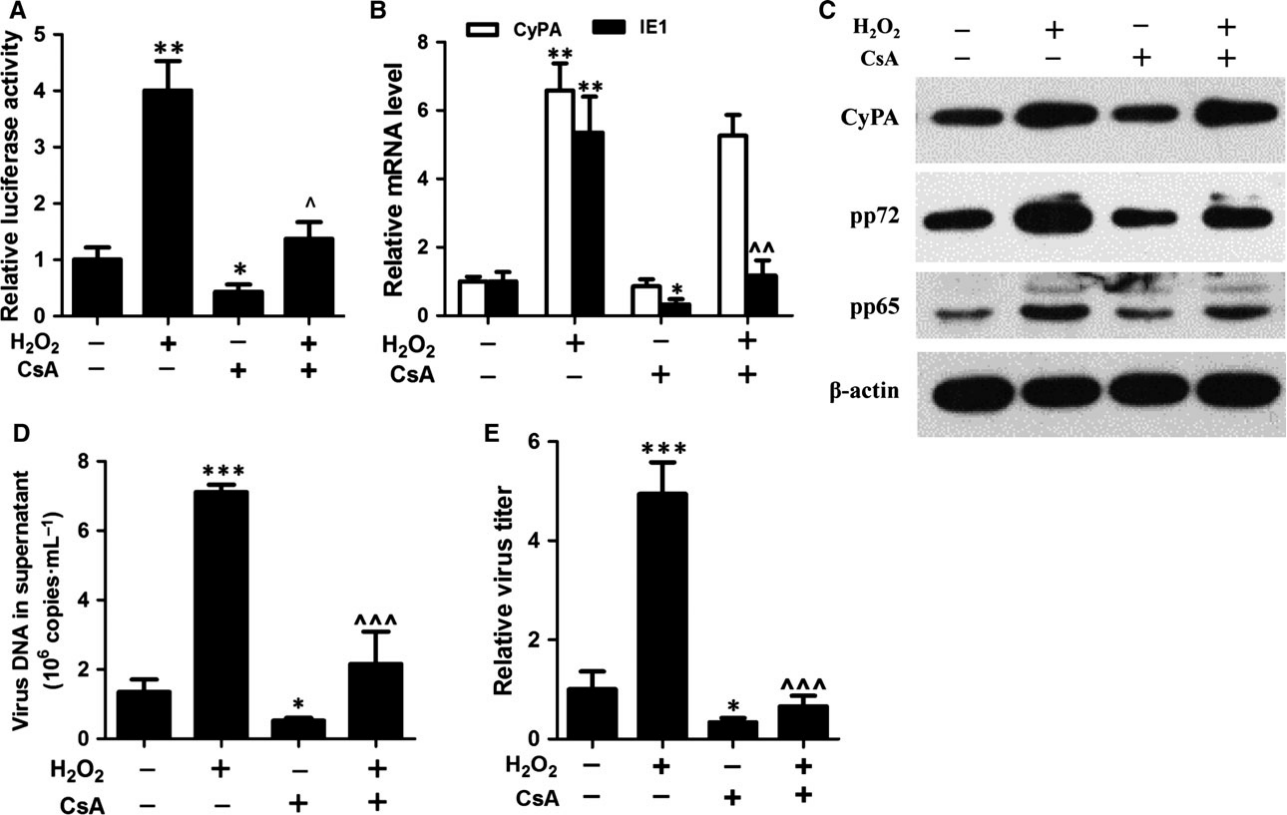
Inhibition of CyPA activity prevents H_2_O_2_‐enhanced human cytomegalovirus (HCMV) replication in human foreskin fibroblast cells. Luciferase activities were detected in HEK 293 cells which were transfected with MIEP‐pGL3 plasmid for 24 h and treated with 1 μm cyclosporine A (CsA) prior to H_2_O_2_ (200 μm) for 12 h (A). Changes of CyPA and IE1 gene expression was evaluated following treatment with CsA (1 μm) in the presence of 200 μm H_2_O_2_ (B). The viral protein expression and viral load in the supernatant were detected by western blotting (C) and quantitative PCR (qPCR) (D) in the presence of CsA (1 μm) prior to H_2_O_2_ (200 μm) treatment for 72 h. Cells treated with CsA (1 μm) in the presence of H_2_O_2_ (200 μm) were infected with HCMV (MOI = 0.5) for 5 days and were analyzed by TCID_50_ assay (E). *P < 0.05; **P < 0.01 or ***P < 0.001 compared with untreated cells. ^*P* < 0.05 or ^^^*P* < 0.001 versus H_2_O_2_‐treated cells.

CyPA is the major intracellular receptor of CsA which is known to bind to and inhibit CyPA activity 26. However, the effect of CsA on the H_2_O_2_‐upregulated CyPA expression has not yet been fully understood. The results presented here demonstrated that CsA exhibited little effect on H_2_O_2_‐upregulated CyPA gene and protein expression (Fig. S3A), as well as H_2_O_2_‐induced ROS (Fig. S3B). To further characterize the influence of CsA on HCMV replication, we evaluated MIEP activity by luciferase assay. The results revealed that H_2_O_2_ stimulated the activity of MIEP, an effect that could be reversed by treatment with CsA (Fig. 3A). Taken together, these results suggested that CsA could hinder the activity, but not the expression of CyPA, thereby resulting in the inhibition of H_2_O_2_‐enhanced viral replication.

To evaluate the antiviral effect of CsA, we evaluated the expression level of viral and host genes. In the presence of H_2_O_2_, viral IE1 mRNA levels were downregulated by treated with CsA, whereas CyPA mRNA levels were not impacted (Fig. 3B). To confirm these findings, we performed western blotting to detect CyPA and viral pp72 and pp65 proteins in infected cells treated for 5 days with CsA. pp72 and pp65 were downregulated by supplementing with CsA, while CyPA expression was unaffected (Fig. 3C).

To examine the effect of CyPA activity on the full cycle of viral replication, real‐time PCR and viral titration were performed to detect viral DNA changes and infectious virion production. Treatment with H_2_O_2_ produced higher viral DNA load and viral titer as compared to the control group, while CsA decreased H_2_O_2_‐upregulated HCMV lytic replication (Fig. 3D,E).

### Effect of CsA on the inhibition of MCMV replication *in vivo*

**Figure 4:**
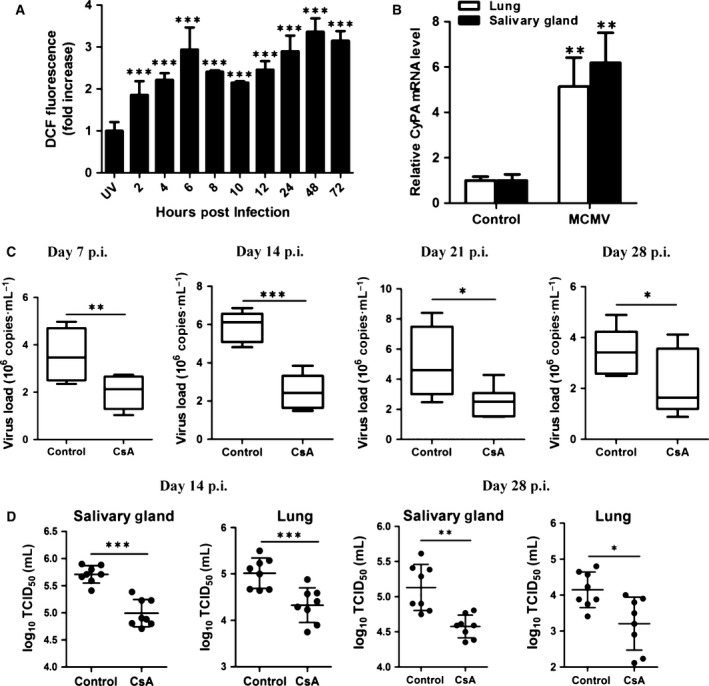
Cyclosporine A (CsA) treatment inhibited MCMV lytic replication *in vivo*. MEF cells in 24‐well plates were infected with either UV‐inactivated MCMV or MCMV (MOI = 0.5) and incubated with 10 μm H2DCF‐DA for 30 min at 37 °C. The H_2_ DCF‐DA fluorescence were detected between infected and uninfected cells at indicated times (A). Fold induction of CyPA expression in infected mice were detected post 14 days MCMV infection (B). Mice were intragastrically treated daily with 10 mg·kg^−1^ CsA, 3 days prior to intraperitoneal inoculation with MCMV (Smith strain, 5 × 10^3^ p.f.u). About 100 μL whole blood from each mouse were examined. Viral DNA loads in blood samples of control (*n* = 8) and CsA‐treated (*n* = 8) mice at indicated days post infection (C). Related infectious viral titer in the salivary gland and in the lung was detected at day 14 and 28 post infection by TCID_50_ assay (D). *P < 0.05; **P < 0.01 or ***P < 0.001 versus untreated mice.

The results mentioned above revealed that CyPA acted as a modulator in H_2_O_2_‐enhanced viral replication *in vitro*. In order to further confirm this effect, we investigated the antiviral effect of CsA on viral replication *in vivo*. The result showed that primary infection of MCMV could induce ROS generation in MEF cells (Fig. 4A). This indicated that the induced ROS might stimulate the expression of CyPA to expand the viral production. After being infected with MCMV for 14 days, higher expression of CyPA was exhibited in the salivary gland and the lung in infected mice (Fig. 4B). Thus, we considered whether CsA could inhibit the viral replication during the primary infection *in vivo*.

Mice were treated with vehicle (olive oil) or 10 mg·kg^−1^·day^−1^ CsA by gavage, 3 days before intraperitoneal inoculation with MCMV (Smith strain, 5 × 10^3^ p.f.u). On days 7, 14, 21 and 28 post infection, viral DNA was extracted from whole blood to explore the impact of CsA treatment. The results demonstrated that CsA treatment significantly decreased the viral DNA load during primary infection (Fig. 4C). Additionally, the viral titration results confirmed the effect of CsA. When compared against controls, CsA treatment resulted in decreased viral production on days 14 and 28 (Fig. 4D). Taken together, these results suggest that CsA treatment might help to prevent CMV replication *in vivo*.

### CyPA is involved in H_2_O_2_‐mediated activation of p38‐MAPK

**Figure 5:**
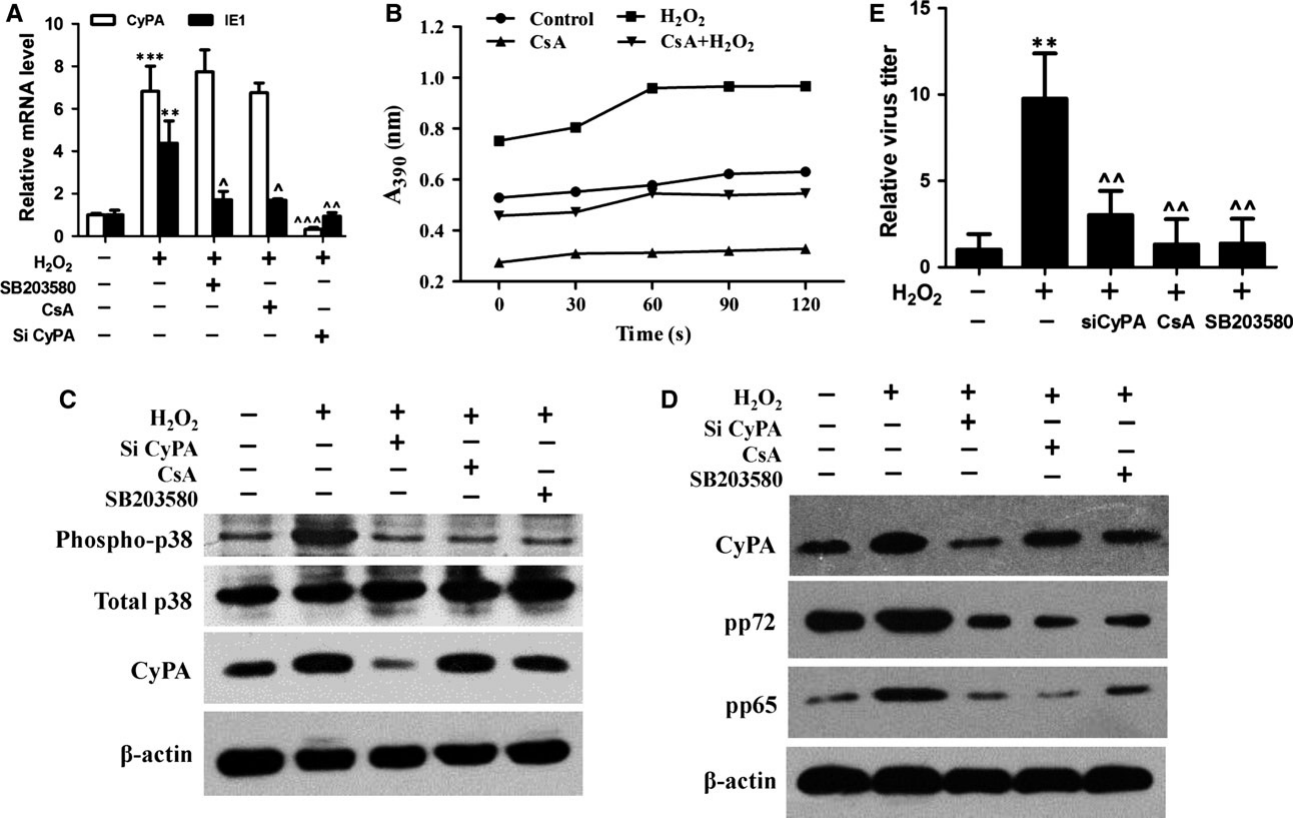
CyPA was involved in the activation of p38‐MAPK pathway during H_2_O_2_‐enhanced human cytomegalovirus (HCMV) replication. Human foreskin fibroblast (HFF) cells and HFF siCyPA cells were treated with SB203580 (10 μm) or cyclosporine A (CsA; 1 μm) 1 h prior to H_2_O_2_ (200 μm). Cells were infected with HCMV at an MOI of 0.5. Real‐time PCR analysis of CyPA and IE1 mRNA levels was performed to compare expression of untreated or H_2_O_2_‐treated cells (A). Cells were treated with CsA (1 μm) prior to H_2_O_2_ (200 μm) for 24 h and then cells were harvested for CyPA activity assay (B). Activation of p38 was detected in HFF cells and HFF siCyPA cells treated with SB203580 (10 μm) or CsA (1 μm) 1 h prior to H_2_O_2_ stimulation. Cells were harvested for western blotting 6 h following H_2_O_2_ treatment (C). Viral proteins were detected in the presence or absence of H_2_O_2_ (200 μm) under treatment with SB203580 (10 μm) or CsA (1 μm) in HFF cells or HFF siCyPA cells (D) post 72 h HCMV infection. Cells treated with CsA (1 μm) or SB203580 (10 μm) in the presence of H_2_O_2_ (200 μm) were infected with HCMV (MOI = 0.5) for 5 days and were analyzed by viral titration (E) **P < 0.01 or ***P < 0.001 versus untreated cells. ^*P* < 0.05; ^^*P* < 0.01 or ^^^*P* < 0.001 compared with H_2_O_2_‐treated cells.

It is known that p38‐mitogen‐activated protein kinase (MAPK) is rapidly and strongly activated by H_2_O_2_ treatment, in a time‐ and dose‐dependent manner. As CsA treatment inhibited H_2_O_2_‐upregulated viral replication, we hypothesized that the CsA‐treatment effect may be related to downstream p38 MPAK pathway activation. As shown in Fig. 5A, inhibition and depletion of CyPA decreased the viral transcription in the context of H_2_O_2_ stimulation, but unlike siCyPA treatment, CsA and SB203580 has no effect on the expression of CyPA. To confirm the effect of CsA on CyPA, the activity of CyPA was detected. The CyPA activity was increased by treatment with H_2_O_2_, while supplementing with CsA decreased the H_2_O_2_‐induced CyPA activity (Fig. 5B). This indicated that inhibition of the H_2_O_2_‐upregulated viral replication may act in a CyPA‐dependent manner. Western blotting revealed that depletion and inhibition of CyPA strongly hindered the H_2_O_2_‐stimulated p38 activation (Fig. 5C) and viral proteins expression (Fig. 5D). In keeping with the RT‐PCR result, the CyPA expression was also not affected by CsA or SB203580, and the viral titer was strongly inhibited by depletion or inhibition of CyPA (Fig. 5E). These results indicate that CyPA is a critical factor in ROS/p38‐MAPK pathway‐regulated HCMV replication.

## Discussion

**Figure 6:**
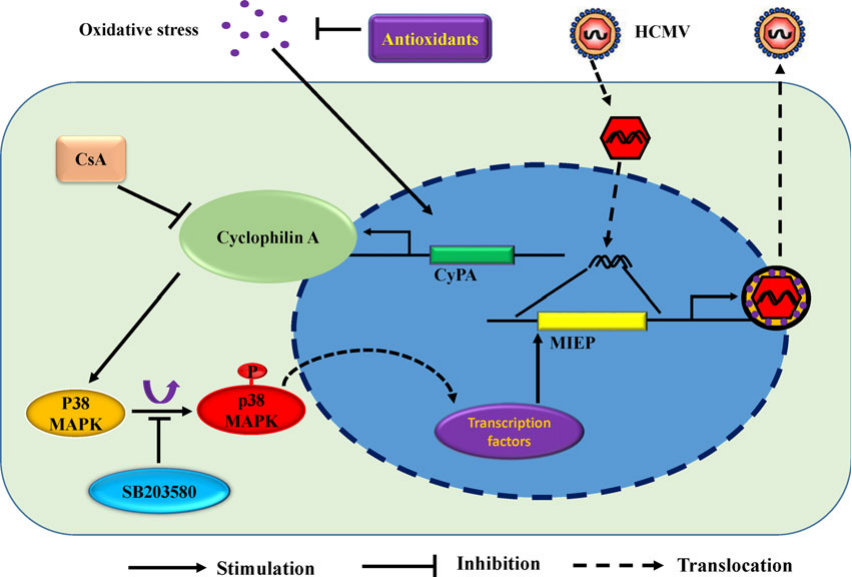
Model for inhibition mechanism of antioxidants, cyclosporine A (CsA) and SB203580. Oxidative stress could enhance human cytomegalovirus (HCMV) replication though ROS/CyPA/p38‐MAPK pathway. However, treatment with antioxidants, CsA or SB203580 to inhibit the molecule involved in this pathway could attenuate the ROS‐upregulated HCMV replication.

Although HCMV replication can be enhanced by treatment with H_2_O_2_ and can be inhibited by antioxidants, the host‐cell molecular interactions involved in H_2_O_2_‐enhanced replication are poorly understood. In this study, we demonstrated that H_2_O_2_ stimulation could enhance CyPA expression in HFF cells, thus resulting in increased HCMV replication mediated by p38‐MAPK pathway activation (Fig. 6).

Cyclophilins are a family of highly conserved and ubiquitous proteins termed immunophilins 27. The most abundant cyclophilin is CyPA 28, which was identified as the main target for the immunosuppressive drug CsA 29, 30. CyPA catalyzes the *cis–trans* isomerization of peptidyl‐prolyl bonds of cytoplasmic proteins, and acts to promote proteins folding and assembly. Previous studies have indicated that CyPA secretion was stimulated by reactive oxygen species (ROS) in vascular smooth muscle cells (VSMC) 12; however, this model has been largely uncharacterized in fibroblasts. In the present study, we demonstrated that CyPA expression is induced by H_2_O_2_ in HFF cells and this effect could be inhibited by the addition of antioxidants.

Several viruses, such as influenza virus, HIV, and HCV, have been reported to induce viral replicatoin in the context of cellular oxidative stress 31, 32, 33. Similarly, the host cellular protein CyPA is also known to be involved in the replication of these viruses 16, 34, 35. It has been reported that HCMV infection induces the generation of ROS minutes after entry into the host cell 4. Furthermore, previous study indicated that oxidative stress could enhance HCMV replication 3, 5. Thus, CyPA may represent a critical factor in mediating the effects of H_2_O_2_‐enhanced HCMV replication. This hypothesis was supported by our results which demonstrated that knockdown of CyPA resulted in a delay in the H_2_O_2_‐upregulated production of HCMV. CyPA appears to play a crucial role in H_2_O_2_‐upregulated HCMV replication in HFF cells.

Cyclosporine A represents a pharmacological means of inhibiting CyPA activity. Studies have shown that CsA can induce high levels of ROS 36. However, CsA supplementation prior to H_2_O_2_ treatment suggested that CsA has no effect on the inhibition of H_2_O_2_‐mediated oxidative stress status and CyPA expression in the present study. This indicates that CsA affects the activity, but not the redox homeostasis and expression of CyPA. CsA supplementation inhibits the MIEP, as well as the viral IE1 gene and protein expression and the production of viral particles in the presence of H_2_O_2_ without affecting the ROS levels or CyPA expression.

Although it has been reported that HCMV could induce multiple means to modulate the redox homeostasis 37, HCMV infection can induce oxidative stress *in vitro* as well as an inflammatory response in primary HCMV infection patients 38, suggesting that CyPA may be induced during HCMV infection. This is may be the reason why silencing CyPA could inhibit the HCMV replication in the absence of H_2_O_2_ 20. Furthermore, this study has demonstrated that CsA could inhibit MCMV replication in neural stem/progenitor cells while it has little impact in MEF cells 19. As an immunosuppressive drug, however, it has been reported that CsA could inhibit MCMV infection *in vivo* 39, but the specific mechanism about this phenomenon is not yet clear. In the present study, the oxidative stress status was induced following infection with MCMV and the CyPA gene expression in mice was also enhanced after infection with MCMV. Consistent with previous results, treatment with CsA inhibited the viral DNA load and titer *in vivo*. Taken together our results suggest that CyPA may play an important role in regulating H_2_O_2_‐upregulated viral replication and indicate that the therapeutic method based on CsA or CsA‐derived chemicals should be an attractive strategy.

Our previous study 3 demonstrated that the p38‐MAPK pathway participates in H_2_O_2_‐upregulation of viral replication. Treatment with CyPA could induce the activation of p38 in KG‐1‐derived DCs 40, while other study showed that silencing CyPA could also enhance the activation of p38 41. Thus, we have no idea about the relationship between CyPA and the activation of p38. In this study, the p38 inhibitor, SB203880, decreased the viral gene transcription, but rarely affected the H_2_O_2_‐induced CyPA expression in HFF. Depleting and inhibiting CyPA, however, reduced p38 phosphorylation, while SB203580 could not affect H_2_O_2_‐induced CyPA protein expression. This indicates that CyPA regulates the activation of p38, whereas p38 has little effect on H_2_O_2_‐induced CyPA expression. These results suggest a relationship between CyPA and the ROS/p38 MAPK pathway during HCMV infection (Fig. 6). However, the mechanism of how CyPA regulates p38 activation needs further study.

Consequently, we provided evidence that CyPA was associated with the regulation of H_2_O_2_‐induced p38 reactivation during HCMV infection. Thus, targeting of ROS/CyPA/p38‐MAPK may be a potential therapeutic or preventive approach in HCMV infection.

## Author contributions

JX designed, performed experiments, and wrote the paper; JD, and SX performed experiments; PM, LPL, XPZ, and BG performed viral titer experiment; JX and JD analyzed the data; YYZ and JBX gave scientific advices and contributed to a deep manuscript revision. All authors contributed substantially to the present work, then read and approved the final manuscript.

## Supporting information


**Fig. S1.** Cell viability was quantitatively evaluated by MTT (3‐[4, 5‐dimethylthiazol‐2‐yl]‐2, 5‐diphenyl tetrazolium bromide) assay.
**Fig. S2.** Silencing CyPA in HFF cells was evaluated using RT‐qPCR and Western blotting to assess protein expression levels of CyPA in HFF mock, HFF siCONTROL (siCTR) and HFF siCyPA cells.
**Fig. S3.** The impact of cyclosporine A (CsA, 1 μm) on the expression levels of CyPA in HFF cells following H_2_O_2_ treatment (200 μm) was assessed by RT‐qPCR and western blotting (A). Staining and the densitometric analysis of 2′,7′‐dichlorodihydrofluorescein diacetate (H2DCF‐DA) fluorescence in the response of H_2_O_2_ and CsA (B).
