# Supplementary material for: Inhibition of cyclophilin A suppresses H2O2‐enhanced replication of HCMV through the p38 MAPK signaling pathway
Source: FEBS Open Bio. 2016 Aug 15;6(9):961–71. doi: 10.1002/2211-5463.12105 (PMC5011495; doi:10.1002/2211-5463.12105)
Supplement: Supplementary file 1 — Fig. S1. Cell viability was quantitatively evaluated by MTT (3‐[4, 5‐dimethylthiazol‐2‐yl]‐2, 5‐diphenyl tetrazolium bromide) assay. Fig. S2. Silencing CyPA in HFF cells was evaluated using RT‐qPCR and Western blotting to assess protein expression levels of CyPA in HFF mock, HFF siCONTROL (siCTR) and HFF siCyPA cells. Fig. S3. The impact of cyclosporine A (CsA, 1 μm) on the expression levels of CyPA in HFF cells following H2O2 treatment (200 μm) was assessed by RT‐qPCR and western blotting (A). Staining and the densitometric analysis of 2′,7′‐dichlorodihydrofluorescein diacetate (H2DCF‐DA) fluorescence in the response of H2O2 and CsA (B). [file FEB4-6-961-s001.pdf]

Figure S1

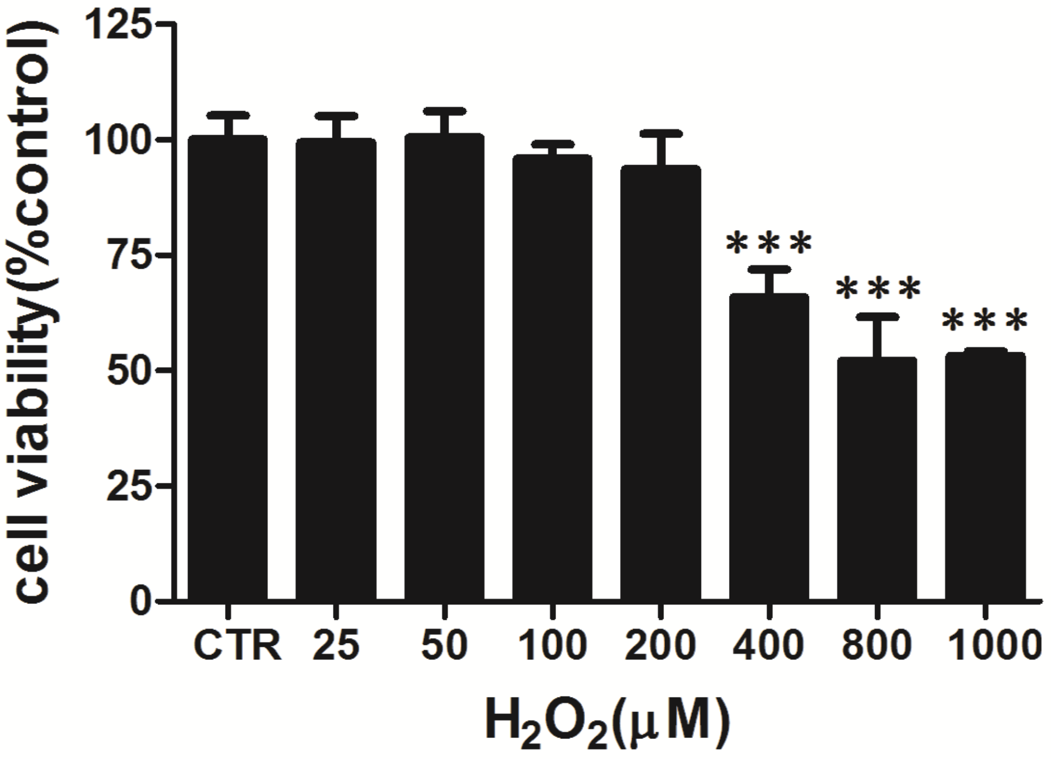

Cell viability was quantitatively evaluated by MTT assay. The confluent HFF cells were pretreated with 25,50,100,200,400,800 and 1000μM H_2O_2 for 24 h. For MTT assay, cells in 96-well plates were incubated with MTT (0.5 g/L, 250 μL per well) at 37℃ for 4 h. Then the medium was carefully aspirated and 100 μL DMSO per well was added to dissolve the blue formazan product. The values of absorbance were measured at 570nm after 30 min. The results of the absorbance of the test wells were expressed as percent of the control wells. Three separate wells for each treatment group were measured.

Figure S2

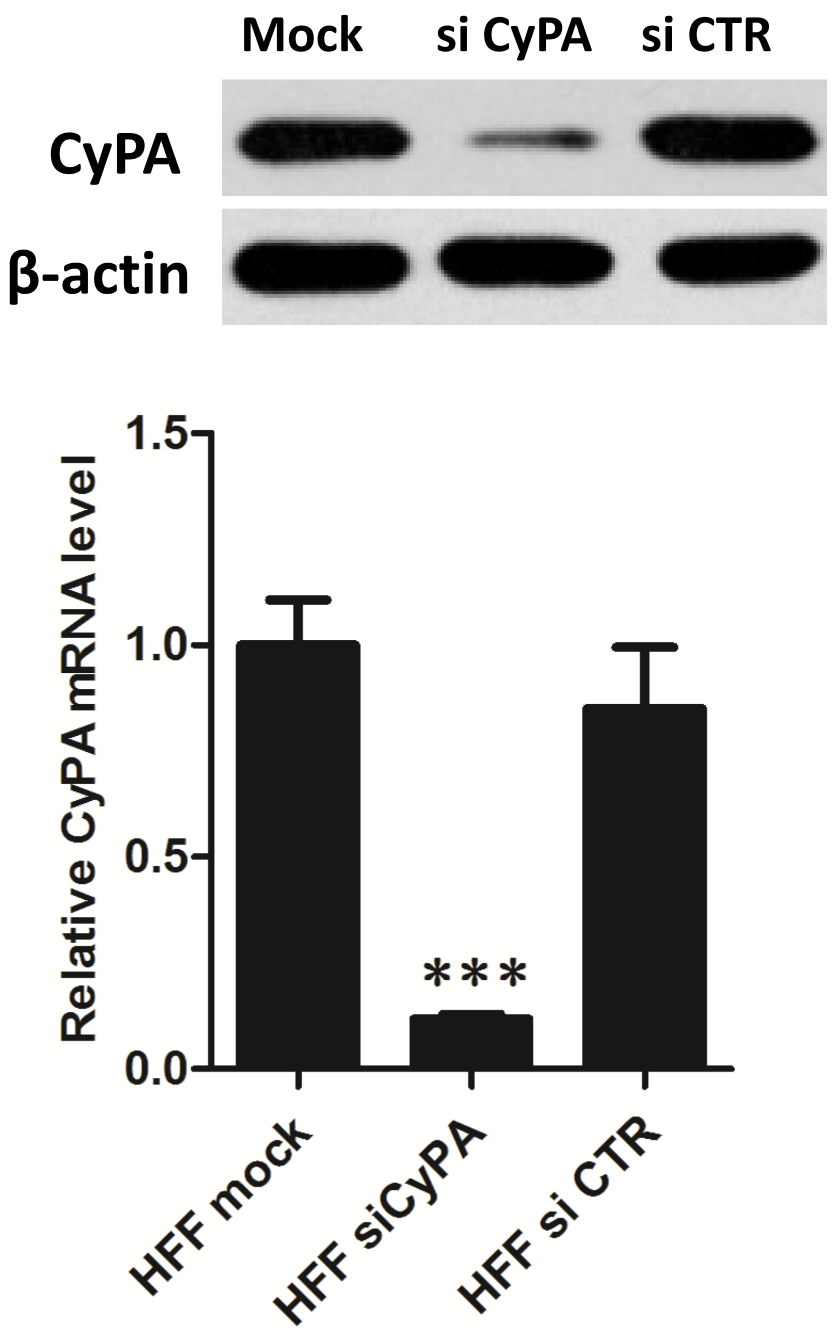

Silencing CyPA in HFF cells was evaluated using RT-qPCR and Western blotting to assess protein expression levels of CyPA in HFF mock, HFF siCONTROL (siCTR) and HFF siCyPA cells.

Figure S3

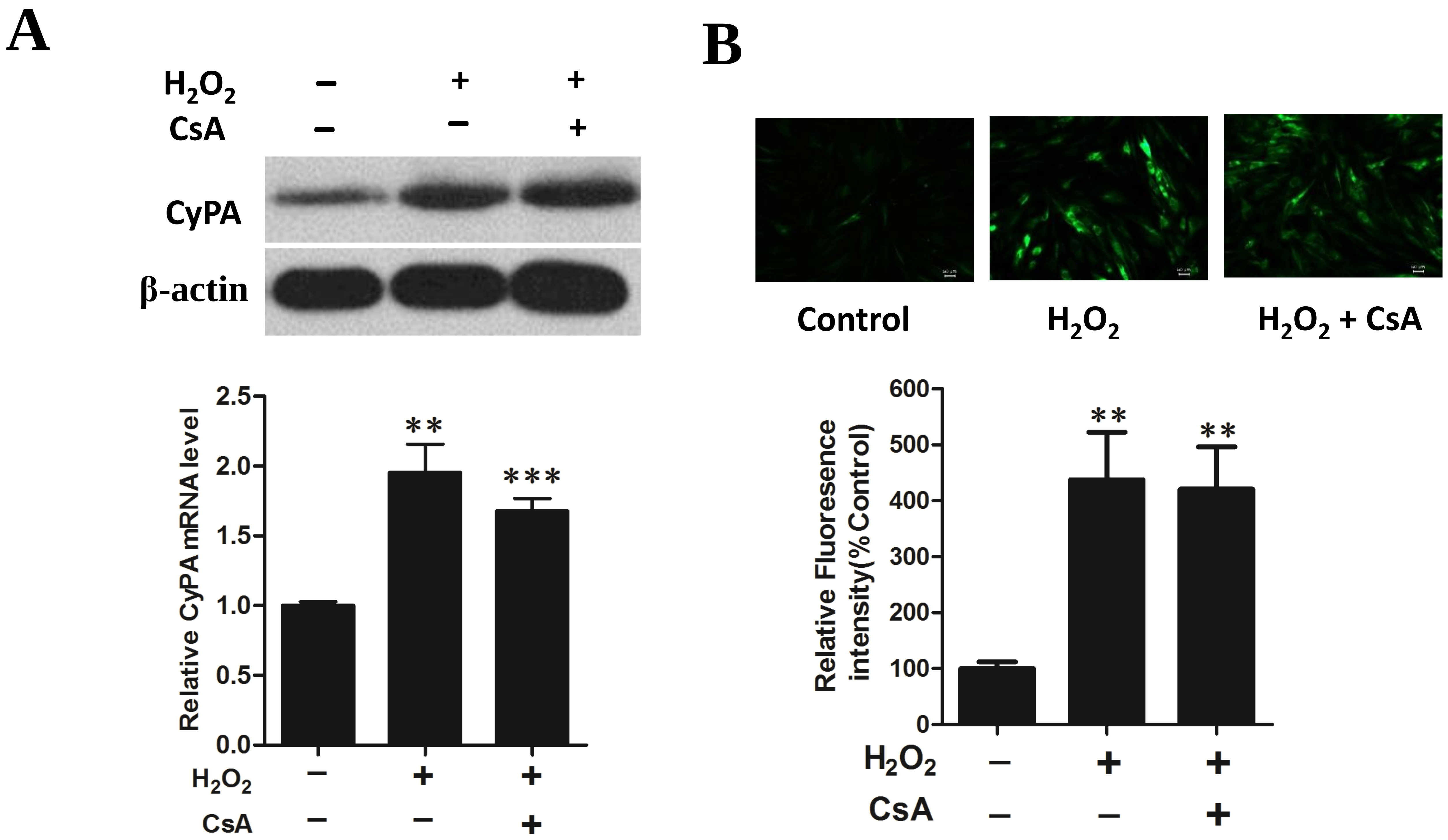

The impact of cyclosporine A (CsA, 1 μM) on the expression levels of CyPA in HFF cells following H_2O_2 treatment (200 μM) was assessed by RT-qPCR and western blotting (A). Staining and the densitometric analysis of 2’,7’-dichlorodihydrofluorescein diacetate (H_2DCF-DA) fluorescence in the response of H_2O_2 and CsA (B).
